# The prognostic value of rectal invasion for stage IVA uterine cervical cancer treated with radiation therapy

**DOI:** 10.1186/s12885-016-2268-3

**Published:** 2016-03-23

**Authors:** Masaru Wakatsuki, Shingo Kato, Hiroki Kiyohara, Tatsuya Ohno, Kumiko Karasawa, Tomoaki Tamaki, Ken Ando, Shintaro Shiba, Tadashi Kamada, Takashi Nakano

**Affiliations:** Research Center for Charged Particle Therapy, National Institute of Radiological Sciences, 4-9-1 Anagawa, Inage-ku, Chiba, Japan; Department of Radiation Oncology, Saitama Medical University International Medical Center, Saitama, Japan; Department of Radiation Oncology, Gunma University Graduate School of Medicine, Maebashi, Gunma Japan; Gunma University Heavy Ion Medical Center, Gunma University, Gunma, Japan; Department of Radiation Oncology, Gunma Prefectural Cancer Center, Ota, Gunma Japan; Department of Radiology, Jichi Medical University, Tochigi, Japan

**Keywords:** Uterine cervical cancer, Stage IVA, Rectal invasion, Radiation therapy, Prognostic value

## Abstract

**Background:**

The prognostic value of rectal invasion is still unclear in stage IVA cervical cancer. The objective of this study is to evaluate patient outcome and prognostic factors in stage IVA cervical cancer treated with radiation therapy.

**Methods:**

A retrospective review of the medical records of patients treated with definitive photon radiation therapy for pathologically proven stage IVA cervical cancer between 1980 and 2010 was performed. Eligible patients for the present study were diagnosed with clinical stage IVA cervical cancer by cystoscopy or/and proctoscopy, and they received definitive radiation therapy consisting of a combination of external beam radiotherapy and high-dose-rate brachytherapy. All patients underwent CT scans of the abdomen and pelvis.

**Results:**

Among the 67 stage IVA patients studied, 53 patients were stage IVA on the basis of bladder invasion, 7 according to rectal mucosal invasion, and 7 because of both bladder and rectal mucosal invasion. Median follow-up of all patients and surviving patients was 19 months (range, 2–235 months) and 114 months (range, 14–223 months), respectively. The 5-year local control (LC), disease-free survival (DFS), and overall survival (OS) rate were 55, 17, and 24 %, respectively. Rectal invasion had significant impact on DFS, but bladder invasion had the opposite effect (*p* = 0.00006 and 0.005, respectively). There were significant differences of LC, DFS and OS rates between patients with and without rectal invasion (*p* = 0.006, 0.00006 and 0.05, respectively).

**Conclusions:**

Patients with stage IVA cervical cancer had poor prognosis, with 5-year survival of only 24 %. Furthermore, in stage IVA, rectal invasion might be a worse prognostic factor than bladder invasion.

## Background

The combination of external beam radiotherapy and intracavitary brachytherapy is considered one of the standard treatments for locally advanced uterine cervical cancer. Stage IVA cervical cancer is defined by the International Federation of Gynecology and Obstetrics (FIGO) and Union for International Cancer Control as a disease directly invading the mucosa of the bladder and/or rectum. There are limited reports on the clinical results for FIGO stage IVA cervical cancer because it represents a small subset of cervical cancer patients, with estimates only around 3.1 % [1]. Three- or five-year overall survival rates for stage IVA disease were reported to be between 21 and 48 % [2–5], and their prognosis is poorer than FIGO II or III disease [3, 6].

On the other hand, according to previous reports in the literature, most of the stage IVA patients were diagnosed based on bladder invasion, and only 1 to 4 patients in any of the studies had rectal invasion [2, 4, 5, 7]. Therefore, the prognostic value of rectal invasion in stage IVA cervical cancer is still unclear. The objective of this study is to evaluate patient outcome and prognostic factors in stage IVA cervical cancer treated with the combination of external beam radiotherapy and intracavitary brachytherapy.

## Methods

### Patients

This retrospective review was performed using the medical records of patients treated with definitive photon radiation therapy for pathologically proven primary invasive cervical cancer at the National Institute of Radiological Sciences, Chiba, Japan, between 1980 and 2010. The eligible patients for the present study were diagnosed with clinical stage IVA cervical cancer by cystoscopy or/and proctoscopy, and biopsy of the bladder or rectal growth was performed for confirmation of stage IVA disease if possible. They received definitive radiation therapy consisting of the combination of external beam radiotherapy and high-dose-rate (HDR) brachytherapy. None of the patients had undergone any surgical procedures including pelvic lymphadenectomy. Pretreatment evaluation was comprised of an assessment of the patient’s history, physical and pelvic examinations by gynecologists and radiation oncologists, cervical biopsy, routine blood cell counts, chemistry profile, chest X-ray, and computed tomography (CT) scans of the abdomen and pelvis. Magnetic resonance imaging (MRI) scans of the pelvis have been performed since 1993. Median age of the patients was 70 years (range, 38–87 years). All patients were staged according to the FIGO staging system, but patients with para-aortic lymph nodes ≥ 1 cm in minimum diameter on CT images were excluded from the study, although patients with enlarged pelvic lymph nodes only were included. The criterion for pelvic lymph node enlargement was ≥ 1 cm diameter on CT images. Cervical tumor size was determined from clinical descriptions, tumor diagrams, CT images and MRI (if available), and was classified into 2 categories (≤6 cm, > 6 cm). A total of 67 patients were admitted to this retrospective analysis. This study was approved by the Ethics Committee of Human Clinical Research of the National Institute of Radiological Sciences in March of 2015, and according to the Declaration of Helsinki in its revised version. The need for informed consent was waived by the Ethics Committee of Human Clinical Research of the National Institute of Radiological Sciences because this study was non-invasive and was based on only medical records.

### Treatment

#### Radiation therapy

Patients were treated with a combination of external beam irradiation and HDR intracavitary brachytherapy or interstitial brachytherapy. External irradiation was delivered with 10 MV photons by using antero-posterior and postero-anterior parallel opposing ports or four-field technique. The common whole pelvic field borders were at the interspace of the L4–5 vertebrae superiorly, at the inferior border of the obturator foramen inferiorly, and at 1.5–2 cm lateral to the bony pelvis. After start of brachytherapy, a central shield was used in the whole pelvic fields. The fraction of external irradiation was mostly 1.8 – 2 Gy midplane tumor-dose daily, four to five fractions weekly to the pelvic lesion. Doses to the whole pelvic fields ranged from 24.0 to 54.0 Gy (median 40.0 Gy), and doses to the total pelvis, consisting of the combined doses to the whole pelvic and central shielding fields, ranged from 44.0 to 61 Gy (median 50.0 Gy). After whole pelvic irradiation, HDR intracavitary brachytherapy by remote afterloading system using iridium-192 or cobalt-60 source was performed. Source arrangement, irradiation conditions, and dose distribution were based on the Manchester system. Point A was defined on X-ray as being 2 cm superior to the external os, and 2 cm lateral from the axis of the intrauterine tandem. HDR brachytherapy was performed at 1 fraction a week, with a standard prescribed dose to Point A of 5–7 Gy per fraction, for a total dose of 1–5 fractions (median: 4 fractions).

### Chemotherapy

In our facility, since 2002 the treatment policy for locally advanced cervical cancer has been concurrent chemotherapy (CCRT) with a chemotherapy regimen of weekly cisplatin (40 mg/m2/week). Concurrent chemoradiotherapy was not performed in patients with insufficient renal function or age over 70 years, so 5 of 17 patients received CCRT after 2002. On the other hand, most of the patients received radiotherapy alone until 2001, and 6 of 52 patients received CCRT with a chemotherapy regimen of bleomycin or peplomycin and mitomycin C. Among all patients, 56 (84 %) were treated by radiotherapy alone and 11 (16 %) by CCRT as well.

### Follow-up

After completion of radiotherapy, patients were followed up every 1–3 months for 2 years, and every 3 or 6 months thereafter. The procedures consisted of a physical examination, routine blood cell counts, chemistry profile, chest X-ray, and CT scan. Suspected persistent or recurrent disease was confirmed by biopsy whenever possible.

### Statistical analysis

Time to recurrence was measured from the date of the start of treatment. The Kaplan-Meier method was used to derive estimates of overall survival (OS), local control (LC) and disease-free survival (DFS) rates. Prognostic values were compared by DFS because the data for this study were from 1980 to 2010, and salvage treatment after recurrence had improved during these 30 years.

Prognostic values for DFS were investigated by log-rank test. Age (≥70 years or < 70 years), tumor size (>6 cm or ≤ 6 cm), rectal invasion (yes or no), bladder invasion (yes or no), parametrium invasion (to pelvic wall or not), vaginal invasion (lower third or not), hydronephrosis (yes or no), pelvic lymph node enlargement (yes or no), concurrent chemotherapy (yes or no), histological type (squamous cell carcinoma or adenocarcinoma) were considered binary variables. Log-rank test was used for statistical analyses of the differences of DFS, LC and OS between the patients with and without rectal invasion. Statistical significance was defined as a *P* value of < 0.05. All statistical analyses were performed using SPSS Statistics version 18.0 (SAS Institute, Tokyo, Japan).

## Results and discussion

Sixty-seven patients with stage IVA were diagnosed and given definitive radiation therapy during this 30-year period at our institution. Patient characteristics are presented in Table 1. Fifty-three patients were stage IVA on the basis of bladder invasion, seven on the basis of rectal mucosal invasion, and seven on the basis of both bladder and rectal mucosal invasion. The median follow-up duration for all patients and surviving patients was 19 months (range, 2–235 months) and 114 months (range, 14–223 months), respectively. Median duration of treatment was 49 days (range, 25 – 59 days). That in patients with and without rectal invasion was 49 days (range, 25 – 59 days) and 49 days (range, 30 – 57 days), respectively. Median total dose of external beam irradiation and HDR brachytherapy with and without central shielding fields along with BED was 91.8 Gy_10_ (range, 68.3 – 117.1 Gy_10_) and 74.4 Gy_10_ (range, 56.3 – 105.1 Gy_10_), respectively. That without central shielding fields in patients with and without rectal invasion was 74.4 Gy_10_, (range, 45.3 – 105.1 Gy_10_) and 76.1 Gy_10_, (range, 56.3 – 90.6 Gy_10_), respectively.

**Table 1:** Patient and disease characteristics (*n* = 67)

Characteristics	No.
Age, y, median (range)	70 (38–87)
Bladder or rectal invasion	
Bladder only	53 (79.1 %)
Rectal only	7 (10.4 %)
Both bladder and rectal	7 (10.4 %)
Parametrium invasion	
To pelvic wall	59 (88.1 %)
Not to pelvic wall	8 (11.9 %)
Vaginal invasion	
To lower third	20 (29.9 %)
Not to lower third	47 (70.1 %)
Hydronephrosis	
Yes	32 (47.8 %)
No	35 (52.2 %)
Pelvic lymph node enlargement	
Yes	27 (40.3 %)
No	40 (59.7 %)
Concurrent chemotherapy	
Yes (CCRT)	11 (16.4 %)
No (radiation therapy alone)	56 (83.6 %)
Histology	
Squamous cell carcinoma	61 (91.0 %)
Adenocarcinoma	6 (9.0 %)
Tumor size	
≤6 cm	22 (32.8 %)
>6 cm	45 (67.2 %)

**Fig. 1:**
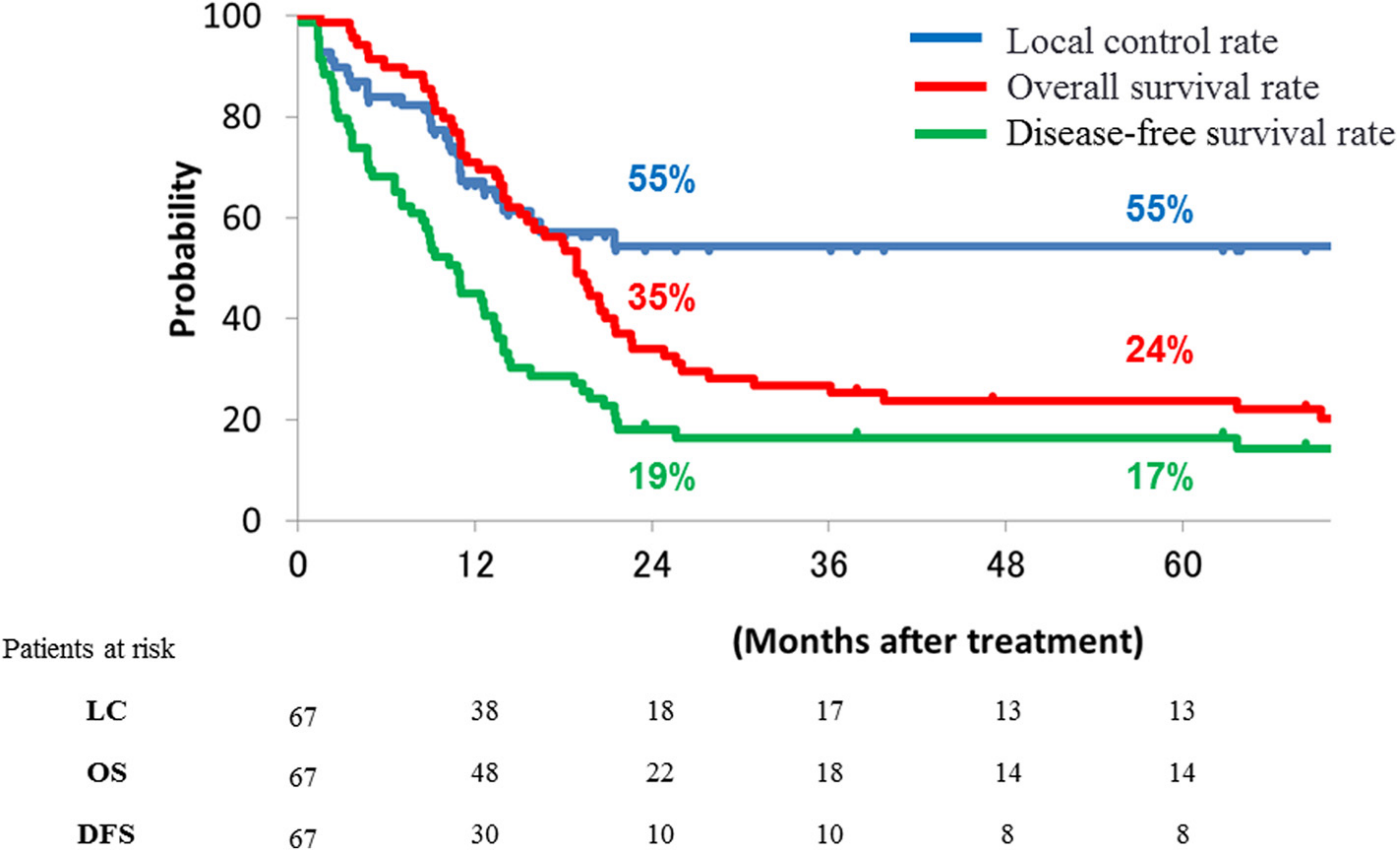
Disease-free survival, local control and overall survival curves; disease-free survival (green line), local control (blue line), and overall survival curves (red line) are shown for all patients

The LC, DFS and OS curves of all patients are shown in Fig. 1. The 5-year LC, DFS, and OS rates were 55, 17, and 24 %, respectively, and the 2-year LC, DFS, and OS rates were 55, 19, and 35 %, respectively. A comparison of prognostic factors for DFS in stage IVA cervical cancer patients is shown in Table 2. Rectal invasion, vaginal invasion, hydronephrosis and pelvic lymph node enlargement showed statistically significant consequences on DFS, while bladder invasion had an opposite effect (Table 2). The patient characteristics with and without rectal invasion are shown in Table 3. The patient group with rectal invasion had a significantly higher rate of lymph node enlargement.

**Table 2:** Comparison of prognostic factors for disease-free survival in stage IVA cervical cancer patients

Prognostic factor	N	Median DFS (mo)	2-year DFS rate (%)	*P* value
Rectal invasion				
Yes	14	3.6	0	0.00006
No	53	13.3	23.6	
Bladder invasion				
Yes	60	11.0	20.9	0.005
No	7	3.4	0	
Parametrium invasion				
To pelvic wall	59	11.0	16.0	0.957
Not to pelvic wall	8	2.4	37.5	
Vaginal invasion				
To pelvic wall	20	5.0	10.0	0.025
Not to pelvic wall	47	12.7	22.4	
Hydronephrosis				
Yes	32	9.0	7.1	0.033
No	35	11.0	28.6	
Pelvic lymph node enlargement				
Yes	27	7.1	11.1	0.036
No	40	13.3	23.7	
Concurrent chemotherapy				
Yes	11	8.7	9.1	0.272
No	56	10.8	20.6	
Histological type				
Squamous cell carcinoma	61	11.0	17.2	0.726
Adenocarcinoma	6	2.6	33.3	
Tumor size				
≤6 cm	22	6.5	27.3	0.689
>6 cm	45	11.0	14.2

**Table 3:** Patient characteristics with and without rectal invasion

Characteristics	Rectal invasion	No rectal invasion (Bladder invasion only)	*P* value
	(*N* = 14)	(*N* = 53)	
Age, y, median (range)	69 (38–80)	71 (38–87)	0.277
Bladder invasion			
Yes	7 (50 %)	53 (100 %)	0.000001
No	7 (50 %)	0	
Parametrium invasion			
To pelvic wall	14 (100 %)	45 (84.9 %)	0.278
Not to pelvic wall	0	8 (15.1 %)	
Vaginal invasion			
To lower third	6 (42.9 %)	14 (26.4 %)	0.386
Not to lower third	8 (57.1 %)	39 (73.6 %)	
Hydronephrosis			
Yes	7 (50 %)	25 (47.2 %)	0.911
No	7 (50 %)	28 (52.8 %)	
Pelvic lymph node enlargement			
Yes	10 (71.4 %)	17 (32.1 %)	0.018
No	4 (28.6 %)	36 (67.9 %)	
Concurrent chemotherapy			
Yes (CCRT)	1 (8.3 %)	10 (18.9 %)	0.517
No (radiation therapy alone)	13 (91.7 %)	43 (81.1 %)	
Histology			
Squamous cell carcinoma	2 (14.3 %)	4 (7.5 %)	0.796
Adenocarcinoma	12 (85.7 %)	49 (92.5 %)	
Tumor size			
≤6 cm	2 (14.3 %)	20 (37.7 %)	0.180
>6 cm	12 (85.7 %)	33 (62.3 %)	

**Fig. 2:**
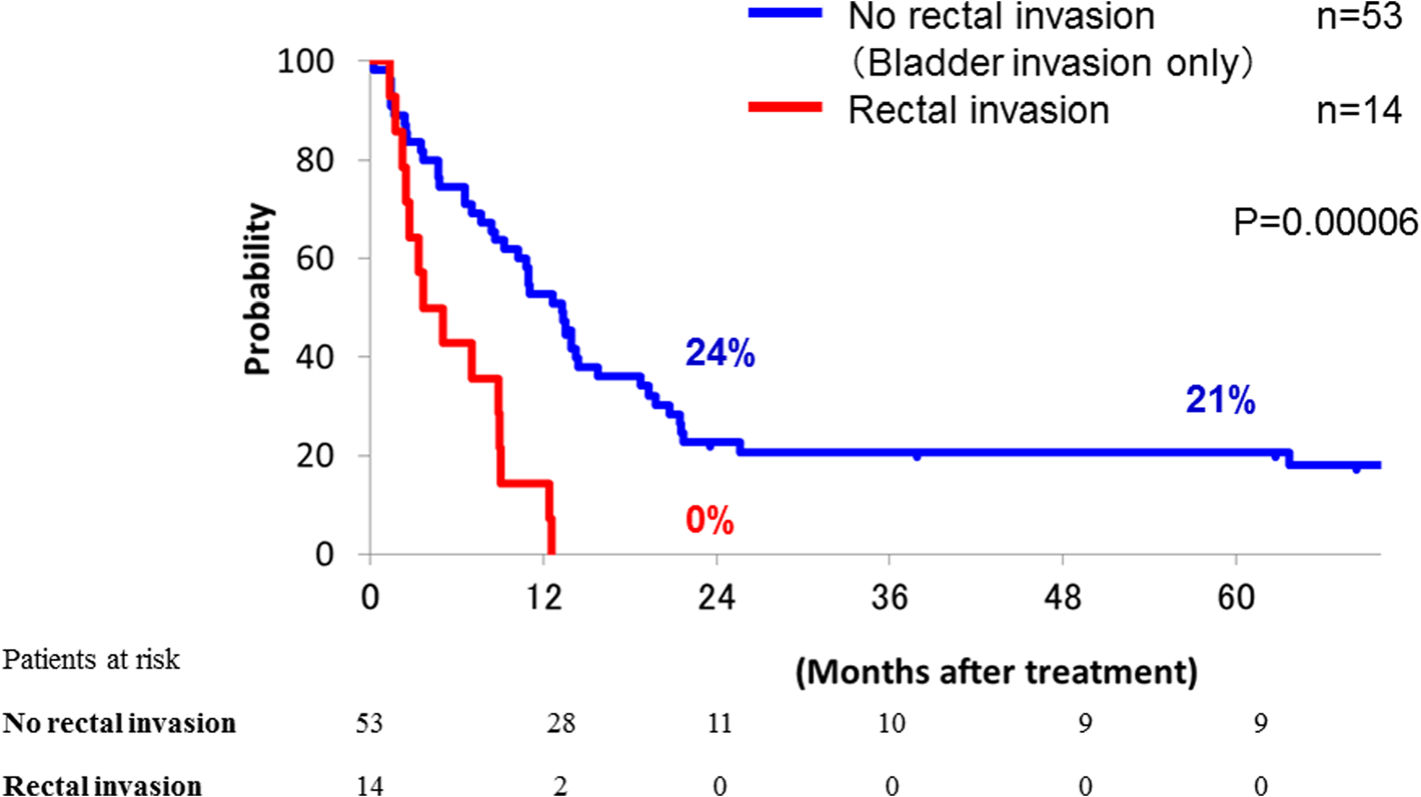
Disease-free survival curves of patients with and without rectal invasion; those of patients with rectal invasion (red line) and without rectal invasion (blue line) are shown

**Fig. 3:**
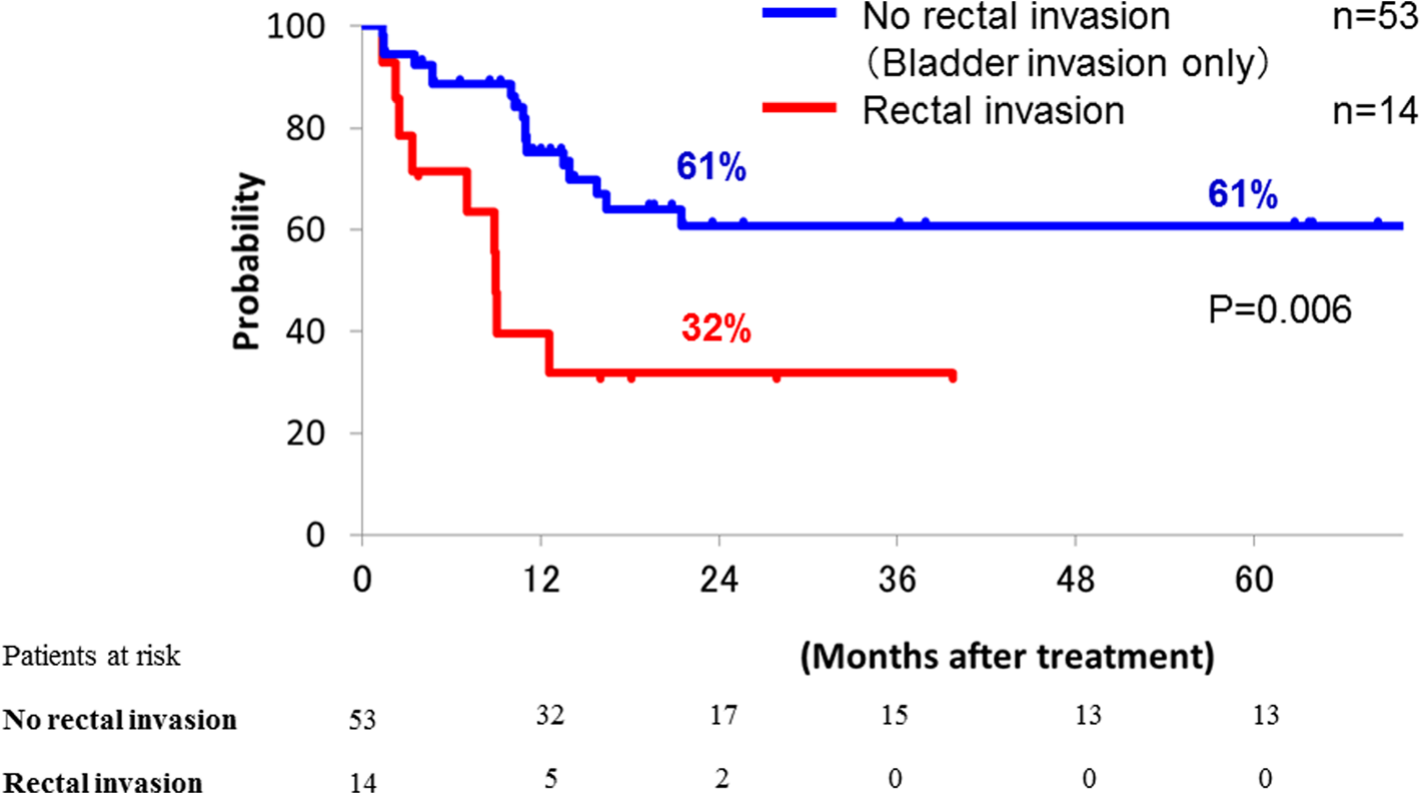
Local control curves of patients with and without rectal invasion; those of patients with rectal invasion (red line) and without rectal invasion (blue line) are shown

**Fig. 4:**
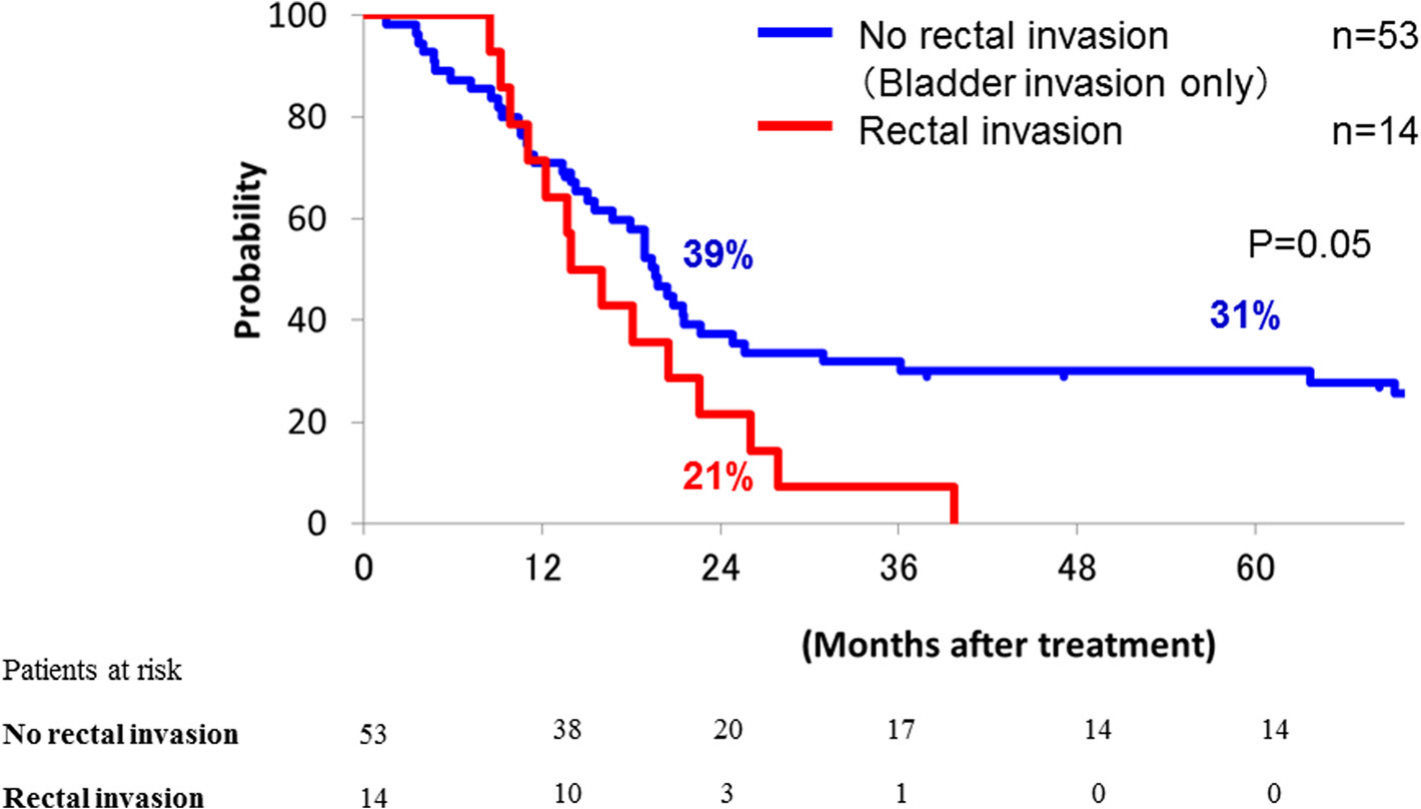
Overall survival curves of patients with and without rectal invasion; those of patients with rectal invasion (red line) and without rectal invasion (blue line) are shown

DFS, LC and OS curves of the patients with and without rectal invasion are shown in Figs. 2, 3 and 4, respectively. All patients without rectal invasion had bladder invasion. There were significant differences of DFS, LC, and OS among the groups (*p* = 0.00006, 0.006 and 0.05, respectively). The 2-year DFS, LC, and OS rates of the patients without rectal invasion were 24, 61, and 39 %, respectively. Those of the patients with rectal invasion were 0, 32, and 21 %, respectively. No patient with rectal invasion survived over 4 years.

The numbers of observed Grade 2 or higher late complications are listed in Table 4. Seven of 67 patients showed late complications of the bladder, 3 of the rectosigmoid colon, and 3 of the small intestine. One patient developed excised perforated intestine or sigmoid colon and vesicovaginal fistula and one patient developed vesicovaginal fistula.

**Table 4:** Grade 2 or higher late complications by RTOG/EORTC scoring scheme

	No.	G2	G3	G4-5
Rect/Sigmoid	67	1	1	1
Bladder	67	4	1	2
Small intestine	67	1	1	1

### Discussion

To the best of our knowledge, this is the first report of the analysis of the prognostic value of rectal invasion. The present study revealed that rectal invasion might be a worse prognostic factor than bladder invasion in Stage IVA cervical cancer after radiation therapy. Rectal invasion, vaginal invasion, hydronephrosis and pelvic lymph node enlargement showed statistically significant effects on DFS in these patients.

Rectal invasion might be a worse prognostic factor than bladder invasion in Stage IVA cervical cancer after radiation therapy. Several researchers reported that the 3-year or 5-year overall survival rates for stage IVA disease were between 21 and 48 % [2–5], although those studies each included only 1 to 4 patients with rectal invasion. In the present study, the 5-year survival rate of patients without rectal invasion was 30 %, a result similar to other reports. However, no patient with rectal invasion survived over 4 years, and rectal invasion showed a significant impact on DFS, as opposed to bladder invasion. DFS differed significantly between patients with and without rectal invasion (*p* = 0.0008). Thus, in Stage IVA cervical cancer, rectal invasion appears to be a poorer prognostic factor than bladder invasion.

Rose et al. and Logsdon et al. reported the significance of hydronephrosis on the outcome of patients with stage IIIB cervical cancer treated with radiation therapy [8, 9]. Cervical cancer extending to the lower third of the vagina has been considered as a factor involved in treatment failure. Kavadi et al. reported that the 5-year survival rate of patients with lower-third vaginal involvement in patients with stage IIIB cervical cancer was only 25 % [10]. Logsdon et al. also reported that poor disease-specific survival was correlated with involvement of the lower third of the vagina [9]. These reports concluded that hydronephrosis and involvement of the lower third of the vagina were poor prognostic factors in patients with stage IIIB cervical cancer. In addition, it is well known that pelvic lymph node enlargement is one of the significant prognostic factors in cervical cancer patients [11, 12]. In the present study, hydronephrosis, involvement of the lower third of the vagina, and pelvic lymph node enlargement showed statistically significant consequences for DFS. Thus, these factors will be poor prognostic factors in patients with stage IVA cervical cancer.

In the current study, 31 % of the patients with bladder invasion and without rectal invasion showed 5 years or longer survival, but no patients with rectal invasion reached 4-year survival, as they all developed local or/and distant failure within 18 months. Therefore, concurrent or adjuvant chemotherapy will be expected to improve the treatment outcome for such patients. Since 2001, on the basis of several randomized trials, the standard treatment for locally advanced cervical cancer, including stage IVA patients, has been CCRT [13–16]. However, there were no significant differences in DFS between patients receiving CCRT and radiation therapy alone in this study. This was obviously related to the fact that only 16 % of the patients received CCRT, as this analysis included the patients before 2000, half of the patients were elderly (median age was 70 years), and many patients had insufficient renal function due to hydronephrosis. Therefore, new treatment techniques, such as intensity-modulated radiation therapy [17], image-guided brachytherapy [18, 19] and carbon ion radiotherapy [20, 21], are expected to improve the treatment outcome of stage IVA cervical cancer.

## Conclusions

The 5-year survival rate of patients with stage IVA disease was only 24 %, so the prognosis was poor. Furthermore, for this group of patients, rectal invasion with or without bladder invasion was a worse prognostic factor than bladder invasion alone in stage IVA. Thus, these patients are in need of even more aggressive therapy.

## Abbreviations

CCRT: concurrent chemotherapy
CT: computed tomography
DFS: disease-free survival
FIGO: International Federation of Gynecology and Obstetrics
HDR: high-dose-rate
LC: local control
MRI: magnetic resonance imaging
OS: overall survival

## References

[1] Quinn MA, Benedet JL, Odicino F, Maisonneuve P, Beller U, Creasman WT (2006). Carcinoma of the cervix uteri. FIGO 26th Annual Report on the Results of Treatment in Gynecological Cancer. Int J Gynaecol Obstet.

[2] Biewenga P, Mutsaerts MA, Stalpers LJ, Buist MR, Schilthuis MS, van der Velden J (2010). Can we predict vesicovaginal or rectovaginal fistula formation in patients with stage IVA cervical cancer?. Int J Gynecol Cancer.

[3] Nakano T, Kato S, Ohno T, Tsujii H, Sato S, Fukuhisa K (2005). Long-term results of high-dose rate intracavitary brachytherapy for squamous cell carcinoma of the uterine cervix. Cancer.

[4] Rose PG, Ali S, Whitney CW, Lanciano R, Stehman FB (2011). Outcome of stage IVA cervical cancer patients with disease limited to the pelvis in the era of chemoradiation: a Gynecologic Oncology Group study. Gynecol Oncol.

[5] Murakami N, Kasamatsu T, Morota M, Sumi M, Inaba K, Ito Y (2013). Radiation therapy for stage IVA cervical cancer. Anticancer Res.

[6] Perez CA, Grigsby PW, Nene SM, Camel HM, Galakatos A, Kao MS (1992). Effect of tumor size on the prognosis of carcinoma of the uterine cervix treated with irradiation alone. Cancer.

[7] Moore KN, Gold MA, McMeekin DS, Zorn KK (2007). Vesicovaginal fistula formation in patients with Stage IVA cervical carcinoma. Gynecol Oncol.

[8] Rose PG, Ali S, Whitney CW, Lanciano R, Stehman FB (2010). Impact of hydronephrosis on outcome of stage IIIB cervical cancer patients with disease limited to the pelvis, treated with radiation and concurrent chemotherapy: a Gynecologic Oncology Group study. Gynecol Oncol.

[9] Logsdon MD, Eifel PJ (1999). Figo IIIB squamous cell carcinoma of the cervix: an analysis of prognostic factors emphasizing the balance between external beam and intracavitary radiation therapy. Int J Radiat Oncol Biol Phys.

[10] Kavadi VS, Eifel PJ (1992). FIGO stage IIIA carcinoma of the uterine cervix. Int J Radiat Oncol Biol Phys.

[11] Wakatsuki M, Ohno T, Kato S, Ando K, Noda SE, Kiyohara H (2014). Impact of boost irradiation on pelvic lymph node control in patients with cervical cancer. J Radiat Res.

[12] Grigsby PW, Singh AK, Siegel BA, Dehdashti F, Rader J, Zoberi I (2004). Lymph node control in cervical cancer. Int J Radiat Oncol Biol Phys.

[13] Whitney CW, Sause W, Bundy BN, Malfetano JH, Hannigan EV, Fowler WC (1999). Randomized comparison of fluorouracil plus cisplatin versus hydroxyurea as an adjunct to radiation therapy in stage IIB-IVA carcinoma of the cervix with negative para-aortic lymph nodes: a Gynecologic Oncology Group and Southwest Oncology Group study. J Clin Oncol.

[14] Morris M, Eifel PJ, Lu J, Grigsby PW, Levenback C, Stevens RE (1999). Pelvic radiation with concurrent chemotherapy compared with pelvic and para-aortic radiation for high-risk cervical cancer. N Engl J Med.

[15] Rose PG, Bundy BN, Watkins EB, Thigpen JT, Deppe G, Maiman MA (1999). Concurrent cisplatin-based radiotherapy and chemotherapy for locally advanced cervical cancer. N Engl J Med.

[16] Chemoradiotherapy for Cervical Cancer Meta-Analysis C (2008). Reducing uncertainties about the effects of chemoradiotherapy for cervical cancer: a systematic review and meta-analysis of individual patient data from 18 randomized trials. J Clin Oncol.

[17] Chen CC, Lin JC, Jan JS, Ho SC, Wang L (2011). Definitive intensity-modulated radiation therapy with concurrent chemotherapy for patients with locally advanced cervical cancer. Gynecol Oncol.

[18] Wakatsuki M, Ohno T, Yoshida D, Noda S-e, Saitoh J-i, Shibuya K (2011). Intracavitary Combined with CT-guided Interstitial Brachytherapy for Locally Advanced Uterine Cervical Cancer: Introduction of the Technique and a Case Presentation. J Radiat Res.

[19] Potter R, Georg P, Dimopoulos JC, Grimm M, Berger D, Nesvacil N (2011). Clinical outcome of protocol based image (MRI) guided adaptive brachytherapy combined with 3D conformal radiotherapy with or without chemotherapy in patients with locally advanced cervical cancer. Radiother Oncol.

[20] Wakatsuki M, Kato S, Ohno T, Karasawa K, Kiyohara H, Tamaki T (2014). Clinical outcomes of carbon ion radiotherapy for locally advanced adenocarcinoma of the uterine cervix in phase 1/2 clinical trial (protocol 9704). Cancer.

[21] Wakatsuki M, Kato S, Ohno T, Karasawa K, Ando K, Kiyohara H (2014). Dose-escalation study of carbon ion radiotherapy for locally advanced squamous cell carcinoma of the uterine cervix (9902). Gynecol Oncol.

